# Response to crizotinib in a lung adenocarcinoma patient harboring EML4-ALK translocation with adnexal metastasis: A Case Report

**DOI:** 10.1097/MD.0000000000004221

**Published:** 2016-07-29

**Authors:** Wenxian Wang, Wei Wu, Yiping Zhang

**Affiliations:** a Department of Chemotherapy; b Department of Pathology, Zhejiang Cancer Hospital; c Key Laboratory Diagnosis and Treatment Technology on Thoracic Oncology, Hangzhou, Zhejiang, China.

**Keywords:** anaplastic lymphoma kinase, case report, crizotinib, nonsmall cell lung cancer, ovarian metastasis

## Abstract

**Background:**

Lung cancer with ovarian metastasis or adnexal metastasis harboring anaplastic lymphoma kinase (ALK) gene translocation is rare. Crizotinib, a novel ALK tyrosine kinase inhibitor, has already shown an impressive single-agent activity in ALK positive lung cancer.

**Methods:**

To summarize the case of clinical data and treatment of a 33-year-old woman with pelvic adnexal metastasis NSCLC.

**Results:**

Histological examination of the tumors showed lung adenocarcinoma. The right lung biopsy tissue and left adnexal mass biopsy tissue both revealed the presence of an ALK rearrangement by Ventana (D5F3) ALK immunohistochemistry assay (Ventana Medical Systems, Roche, Inc., Tuscon, AZ). The patient experienced a remarkable tumor response to crizotinib treatment.

**Conclusion:**

Although the adnexal location is an uncommon metastasis site from lung cancer, oncologists should be aware of the possibility of such metastasis for female patients with ALK rearrangement NSCLC. Considering this remarkable response, we conclude that the presence of adnexal metastasis in NSCLC patients with ALK rearrangement should be attentive.

## 1 Introduction

Lung cancer is the leading cause of cancer death worldwide. Its morbidity and mortality are increasing in both developed and developing countries.^[1,2]^ It is a very rare metastatic site in the adnexal metastasis from lung cancer. To most female patients, the adnexal tumor is from ovarian cancer, and the ovary is an uncommon metastasis from lung cancer.^[3]^ Specific molecular mutations in certain lung cancer cases have provided excellent opportunities for the development of new targeted therapies. Anaplastic lymphoma kinase (ALK) positive nonsmall cell lung cancer (NSCLC) is found in 2% to 7% of patients.^[4]^ Crizotinib is a first small molecule tyrosine kinase inhibitor to ALK, and it has been approved for the first-line treatment of ALK rearranged NSCLC patients. Comparing with first-line standard chemotherapy, the objective response rate to crizotinib is about 70% and its median progression-free survival is 10 months.^[5]^ In our report, we first present a successful treatment case of an ALK rearrangement adnexal metastasis from lung adenocarcinoma patient with crizotinib.

**Table:**
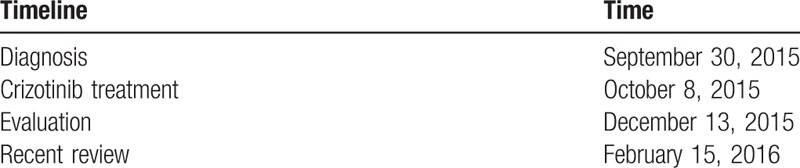

## 2 Case report

### 2.1 Patient information

A 33-year-old woman who was a never-smoker was found pulmonary mass and adnexal mass by health examination in September 2015.

### 2.2 Diagnostic assessment

**Figure 1:**
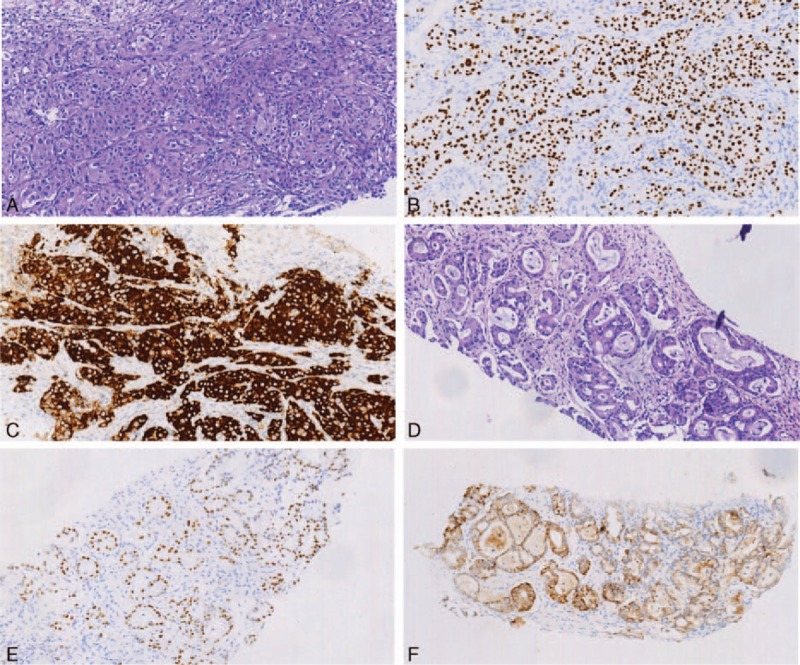
(A) Hematoxylin and eosin (H & E) showed an adenocarcinoma cell carcinoma from lung tumor (H & E ×200). (B) Immunohistochemical (IHC) analysis revealed that the lung tumor cells were positive for thyroid transcription factor-1 (TTF-1) (×200). (C) IHC analysis of the lung tumor with the antibody-D5F3 revealed strong anaplastic lymphoma kinase (ALK) positivity. (D) H & E showed an adenocarcinoma cell carcinoma from pelvic adnexal tumor (H & E ×200). (E) IHC analysis revealed that the pelvic tumor cells were positive for TTF-1 (×200). (F) IHC analysis of the pelvic adnexal tumor with the antibody-D5F3 revealed ALK positivity (×200).

**Figure 2:**
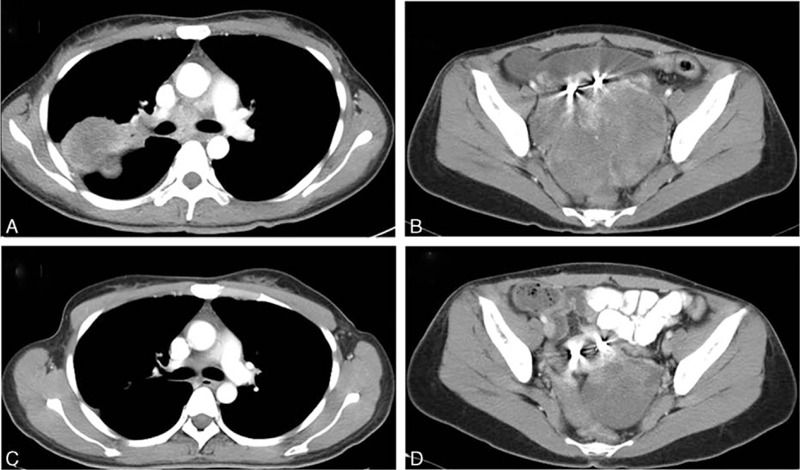
(A) Computed tomography (CT) revealed a mass at superior lobe of right lung and mediastinum lymph nodes enlargements. (B) CT revealed a mass at pelvic adnexal area. (C) CT revealed an excellent tumor response in the right lung after 2 months of treatment with crizotinib. (D) CT revealed an excellent tumor response in pelvic adnexal area after 2 months of treatment with crizotinib.

Computed tomography (CT) scan revealed a 5.8 × 4.5 cm mass at superior lobe of right lung and pulmonary hilar, mediastinum, and bilateral supraclavicular lymph nodes enlargements (Fig. 2A), and CT showed a 7.9 × 6.9 cm mass at adnexal area (Fig. 2B). A pathological diagnosis of adenocarcinoma was performed using CT-guided percutaneous lung needle biopsy. Hematoxylin and eosin (H & E) staining showed a typical morphology of adenocarcinoma cell (Fig. 1A). Immunohistochemistry (IHC) analysis demonstrated positivity in cytokeratin (CK) 7, napsin A, and thyroid transcription factor-1 (TTF-1) (Fig. 1B), although the cells were negative for CK5/6 and p63. She also underwent the adnexal mass biopsy. Combining with H & E (Fig. 1D) and IHC (Fig. 1E), we thought the adnexal mass was metastatic tumor from lung adenocarcinoma but not the ovarian cancer (T3N3M1, stage IV). Lung tumor tissue was wild-type of epidermal growth factor receptor variants that were detected by ARMS (AmoyDx, Xiamen, Fujian, China). Ventana (D5F3) ALK IHC assay (Ventana Medical Systems, Roche, Inc.) analysis of the right lung biopsy tissue (Fig. 1C) and left adnexal mass biopsy tissue (Fig. 1F) both revealed the presence of ALK positive.

### 2.3 Interventions

We treated the patient with crizotinib in October 2015. After 2 months of therapy, CT scan showed dramatic tumor response with the right lung (Fig. 2C) and pelvic tumor size (Fig. 2D). We measured disease as assessed according to the Response Evaluation Criteria in Solid Tumors, version 1.1. The patient was considered as partial response to crizotinib. The patient didn’t experience treatment-related adverse reactions including vision disorders, nausea, diarrhea, vomiting, or QT-interval prolongation.

### 2.4 Follow-up and outcomes

So far, after 4 months, the disease was still stable.

## 3 Discussion

In NSCLC, ALK rearrangement is associated with distinct clinicopathological features, including young age, absent or minimal smoking history, and adenocarcinoma histology.^[6]^ Our case demonstrated an ALK rearrangement adenocarcinoma who was a young, female, never-smoker patient. As is well known, the proportion of ovarian metastasis from lung cancer is very low, it accounts for 0.3% to 0.4% of ovarian metastasis,^[7]^ and Irving et al^[7]^ demonstrated that women lung adenocarcinoma with metastatic ovarian were found to have a mean age of 46 years. Fujiwara et al^[8]^ reported a 39-year-old and Lee et al^[9]^ reported a 54-year-old patient of bilateral ovarian metastasis of NSCLC with ALK rearrangement, respectively. However, they didn’t receive crizotinib treatment. Our case is the first report of crizotinib effective for ALK-positive adenocarcinoma with adnexal metastasis. Furthermore, crizotinib is sensitive to the metastasis in the adnexal metastasis from lung cancer. So, to ensure appropriate management for young female patient harboring ALK fusion, oncologists should be aware of the presence of adnexal or ovarian metastasis particularly. Moreover, clinical features might be useful to help identify a subset of patients who need an ALK testing (e.g., patients with young age, no smoking history, and presence of pelvic metastasis as the case in this report). However, in our case, the patient did not experience a surgery and couldn’t clear the definite regions of adnexal mass. In addition, because of insufficient samples, we didn’t detect ALK fusion gene in adnexal tumor tissue by fluorescence in situ hybridization (FISH) assay again. There are limitations. Further research is needed to analyze the correlation between ALK rearrangement and the metastatic behavior to the ovary or the adnexal area.

In our case, we used Ventana (D5F3) assay to detect the ALK expression. We didn’t use FISH assay to confirm the ALK fusion. Numerous studies indicate that IHC, under the appropriate conditions, is sensitive and specific for determination of ALK protein expression.^[10]^ Nowadays, Food and Drug Administration and China Food And Drug Administration both approved the Ventana (D5F3) to the screen for ALK rearrangements in the evaluation of NSCLC. In our report, the patient experienced a remarkable tumor response to crizotinib. So, we think that the ALK IHC (D5F3) assay is sensitive in the use of ALK test in lung adenocarcinoma.

## 4 Conclusion

It is very important for distinguishing between primary ovarian cancer and metastatic carcinoma from lung cancer because the treatments and prognosis are distinctly different for them. Nowadays, we pay close attention to the precision medicine for cancers, particularly in lung cancer. To make greater benefit for patients, we should be aware of the possibility for such metastasis when the patient has 2 parts of tumors with ALK-positive and do appropriate treatment strategy.

## Acknowledgments

Informed consent: The patient signed informed consent for the publication of this case report and any associated images. This study was approved by the ethics committee of Zhejiang Cancer Hospital.
